# Monkeypox during COVID-19 era in Africa: Current challenges and recommendations

**DOI:** 10.1016/j.amsu.2022.104381

**Published:** 2022-08-18

**Authors:** Osaretin Christabel Okonji, Emeka Francis Okonji

**Affiliations:** School of Pharmacy, Faculty of Natural Science, University of the Western Cape, Cape Town, South Africa; School of Public Health, University of the Western Cape, Cape Town, South Africa

**Keywords:** Monkeypox, Outbreak, COVID-19, Africa, Public health

## Abstract

In May 2022, monkeypox virus (MPXV) outbreak was confirmed amid the coronavirus disease 2019 (COVID-19) pandemic in many parts of the world including Africa. This is the largest outbreak since monkeypox (MPX) was first detected in humans in 1970. The MPX outbreak in Africa is an added burden to the fragile healthcare systems that are already overburdened with several reoccurring epidemics. Although several efforts are in place to effectively contained the outbreak. Several measures such as improved surveillance and diagnostic are necessary to contain the spread of the disease in Africa. This commentary highlights the challenges with the MPX outbreak in Africa and discusses the measures that can be taken to limit the spread of the disease, particularly in high-risk countries.

## 1 Introduction

The current outbreak of monkeypox virus (MPXV) is a public health challenge that is currently observed in several parts of the world. This current outbreak of monkeypox (MPX) is the largest and most widespread outbreak globally. Since the beginning of 2022 more than 28,000 cases have been reported from 88 countries worldwide [1]. Currently 301 cases have been reported in the Africa region and five deaths, most deaths occurred in the Africa region [2]. The World Health Organization (WHO) declared the multi-country MPX outbreak a global public health emergency on July 23, 2022 [2]. Eleven countries are affected in the Africa region accounting for approximately 12% of all reported cases [3]. It is the first time that local transmission of MPX has been reported in non-endemic countries without any link to endemic countries in West and Central Africa that have previously reported MPX outbreak. The source of the current outbreak is unknown till date. The context and transmission of the viral disease is confusing due to the abnormally high incidence of human-to-human transmission observed during this outbreak. The recent spread of MPX in non-endemic regions is a worrying trend that needs urgent attention, because the outbreak is spreading very fast to other countries.

Monkeypox was detected the first time in 1970 in the Democratic Republic of Congo (DRC), since then the viral disease is endemic to DRC and there have been numerous outbreaks in Central and West Africa. Since 1970, human cases of MPX have been reported in 11 countries in Africa: Benin, Cameroon, Central African Republic, Cote d’ Ivoire, DRC, Gabon, Liberia, Nigeria, Republic of the Congo, Sierra Leone and South Sudan [4]. Although the true burden of MPX is unknown. Two distinct clades are responsible for MPX outbreak in central and West Africa: the Congo Basin and West Africa clade [5]. Human infections with the Congo Basin Clade causes severe disease with a case fatality rate (CFR) of 10.6% in relative to the West Africa clade that causes less severe disease with a CFR of 3.6% [6].

Monkeypox is a viral zoonotic disease caused by MPX virus-a member of the Orthopoxvirus genus in the family Poxviridae, characterized by excessive rash or lesions, severe headache, fever, lymphadenopathy, lack of energy and a CFR of about 3–6% [4]. It is transmitted through direct contact with body fluids, lesions, respiratory droplets and contaminated items such as bed sheets [6]. The incubation period generally ranges from six to 13 days, although it can also range from five to twenty-one days [6]. Numerous animal species are known to be susceptible to MPXV, but the exact reservoir is uncertain. Additionally, the consumption of inadequate cooked meat and other animal products of infected animals is also a potential risk factor for acquiring MPX [6].

According to the most recent data on MPX in Africa, the number of cases has increased significantly since April 2022 compared to the same period in 2021. Since the beginning of 2022, 2,867 cases and 103 deaths (CFR: 3.6%) of MPX have been reported from nine endemic and two non-endemic African countries [7]. However, since the beginning of 2020 during the COVID-19 pandemic to date the Africa region has documented 12,457 cases and 365 deaths (CFR 2.9%) of MPX; in year 2020–7,376 MPX cases and 203 deaths (CFR2.8%); in year 2021 3,050 MPX cases with 87 deaths (CFR2.9%); and in 2022–2,031 cases with 75 deaths (CFR 3.1%) [3]. These numbers are most likely underestimated, and the increase in cases are mainly observed in the DRC and Nigeria. Since the last three years (2020–2022), the MPX outbreak in the Africa region continues to spread from one country to another with not much international concern. These statistics from these countries shows that this viral disease requires urgent attention in sub-Saharan Africa even amid the pandemic.

Numerous factors including the waning of orthopoxvirus-related herd immunity from prior smallpox vaccination and the increased human contact with MPX related animal reservoirs accelerated by deforestation, trade, animal husbandry and climate change are responsible for reemergence of the viral disease in African countries [5]. The monitoring of potential animal reservoirs, such as rodents, but also of novel transmission routes, must be considered. Additionally, the Africa region is facing increasing risk of outbreaks caused by zoonotic pathogens, such as MPXV which originated from animals and then changed specie and infected humans. According to an analysis by the WHO the number of zoonotic outbreaks in the Africa region increased by 63% between 2012 and 2022, compared to 2001 to 2011(8). The analysis shows that between 2001 and 2022, 1843 substantiated public health events were recorded in the African region, and 30% of these events were zoonotic disease outbreaks. Although these numbers have increased over the last two decades, there was a particular spike in 2019 and 2020 when zoonotic pathogens accounted for approximately 50% of public health events. Ebola Virus Disease and other viral hemorrhagic fevers accounts for almost 70% of these outbreaks; with monkeypox, dengue fever, anthrax, plague, and a range of other diseases accounting for the remaining 30% [8].

The current ongoing outbreak of MPX mainly affects men identified as gay, bisexual and other men who have sex with men and those who have reported sex with one or multiple partners in other regions excluding the African region [2]. Preliminary data from the World Health organization shows that 99% are males with median age of 36 years (Interquartile range 31–43) and males between 18 and 44 years of age are disproportionately affected by this outbreak of MPX since they account for 77% of cases [2]. An estimated 98% are gay, bisexual or men who have sex with men, and 41% of cases with known HIV status were HIV-positive [2,9]. About 319 cases are health care workers, most were infected in the community, however investigations are ongoing to check if whether infections in health care workers is due to occupational exposure [2]. Available specific data of MPX from the African region shows that 53% were male and a median age of 17 years, making case demographics in Africa similar to recent outbreaks but significantly different from other regions [2,8].

In Africa, endemic countries such as Cameroon, Central African Republic, Congo, DRC, Liberia and Nigeria have recently reported MPX outbreaks. Apart from the six countries in Africa with a history of human transmission, MPX has also been reported in South Africa, Morocco, Benin, Sudan and Ghana, these countries have not previously reported any human cases [1]. South Africa confirmed the viral disease in three patients, with no travel history in two patients, which suggests a high possibility of local transmission. Ever since 2003, import or travel related spread of MPX beyond the Africa continent has occasionally resulted in outbreaks. Monkeypox is becoming a global public health issue since it is not only affecting endemic countries in Central and West Africa, but other part of the world. The spread of MPX to other countries in Africa and other regions where cases have never been identified before is a worrying sign.

Although MPX is self-limiting, but may be severe in certain individuals, like pregnant women, children and immunocompromised individuals [6]. Children who were infected with MPX are known to have severe disease and higher fatality rate in relative to adults [5]. There is also an assumption that immunocompromised persons particularly HIV-positive people have increased risk of developing severe form of the viral disease, but there is scanty data to confirm this. A study that described the 20I7-2018 MPX outbreak in Nigeria found that four out of seven people who died from the viral disease were HIV-positive [5]. Although these patients were not on antiretroviral therapy before infected with MPXV. The spread of MPXV among children, pregnant women and immunocompromised individuals is a worrying concern.

The World Health Organization (WHO) reported 1315 cumulative cases of MPX in endemic countries between 15 December 2021 and 1 May 2022, with the DRC reporting the highest number of cases 1238 cases between 1 January to 1 May 2022 and 57 deaths [6]. The DRC has been reporting increasing number of cases since 1990 (511 cases) through 2000–2019 (more than 28,000 cases) [10] Nigeria reported 46 cases between the 1st of January to 30 April 2022 [6], since in the 1970s Nigeria has reported increasing numbers from three to 181 cases in 2017–2019 [10]. Nigeria continues to experience large outbreaks with more than 500 suspected cases and 200 confirmed cases and a case fatality ratio of approximately 3% ever since 2017 [4]. Cameroon reported 26 cases between 15 December to 22 February 2022 and the Central African Republic reported 6 cases between 4 March to 10 April 2022 [6]. Poor sanitation and hygiene, fragile health care systems, humanitarian crisis, and climate conditions accounts for the disproportionate burden of MPX in Africa [4].

### 1.1 Current effort and challenges

One of the major challenges with regards the MPX outbreak is the weakness of surveillance and laboratories in African countries. At present, a large number of suspected cases exist in the African region, the greatest burden of MPX is in the DRC and Nigeria, together these two countries account for 92% of all suspected cases. With over 80% from the DRC, underscoring the urgent need for enhanced diagnostic capacity [11]. The case of the DRC shows how complex the challenges are and the country has recorded almost 90% of deaths (65 in total) but only 10 laboratory-confirmed cases of MPX since January this year [12]. Although the surveillance and laboratory diagnostic capability implemented by African countries during the COVID-19 pandemic could form the backbone of the fight against MPX, however, many African countries lack skilled medical personnel and chemicals needed for MPX testing [11]. So far, African Union Member States still lack the essential tools necessary for pandemic preparedness and response, including diagnostics, treatments and vaccines [3].

Another challenge is the scarce MPX vaccines, although the smallpox vaccine protects against MPX, but the world stopped using it in the 1970s, shortly before smallpox was declared eradicated. Consequently, there are numerous individuals who are now susceptible to MPX. In the United States and United Kingdom, a vaccine produced by Bavarian Nordic that was approved for MPX is been offered to high risk contact, but this vaccine is not available in Africa.

Although the African continent has reported not much MPX cases as compared to the European region and the United States in this current outbreak, however suspected cases remain undetected in the Africa region. Though there are several efforts by the World Health Organization (WHO) and Ministry of Health in controlling the spread of the viral disease including response activities and increased surveillance, the rapid spread of the viral disease is a serious concern. The global outbreak of MPX has again highlighted global health inequities and has also brought much needed attention of MPX in Africa.

It is very concerning that the MPX outbreak is occurring simultaneously in regions that are currently experiencing numerous outbreaks, including Marburg virus and COVID-19 [[13], [14], [15]]. All of these cause a strain on the fragile health care systems. Several outbreaks of infectious diseases continue to pose a threat to public health, in particular in the African region, however there has been limited attention to strengthening health surveillance systems [15,16].

### 1.2 Recommendations and conclusions

Although not much MPX cases have been reported in Africa as compared to other regions in this current outbreak, it is a major public health concern with scarce vaccine coverage due to limited vaccine supply. It is crucial to promote epidemiology, integrated surveillance programs and laboratory diagnosis particularly in the affected countries in Africa. Increased contact tracing is crucial to the identification of cases. Increased communication and awareness, early diagnosis, active disease surveillance, are crucial in controlling the spread of this viral disease. Health care professionals, laboratory personnel and vulnerable populations should be prioritised for vaccination when it becomes available.

The potential for further transmission of MPXV in Africa and other settings in the coming months is high due to the poor diagnostic and surveillance in many settings. If appropriate measures are put in place, this current outbreak could easily be contained. The current global spread of MPX should be a catalyst for understanding and overcoming this disease in Africa. Currently, more partners and funds are needed to contain the outbreak. Prompt and effective interventions are crucial at this stage so the health care systems are not overwhelmed, which can occur if the MPX outbreak and other outbreaks spread rapidly and extensively. The government and stakeholders should work together and ensure that vulnerable populations are vaccinated, with particular efforts made to ensure timely access to vaccines.

## Provenance and peer review

Not commissioned, externally peer reviewed.

## Please state any sources of funding for your research

None.

## Ethical approval

Not applicable.

## Consent

Not applicable.

## Authors contribution

OCO came up with the idea and conceptualization of the study. OCO and EFO drafted the manuscript. OCO and EFO, made the critical review of the manuscript. Both authors read and approved the final manuscript.

## Registration of research studies

1. Name of the registry: Not applicable
2. Unique Identifying number or registration ID: Not applicable
3. Hyperlink to your specific registration (must be publicly accessible and will be checked): Not applicable

## Guarantor

Osaretin Christabel Okonji, School of Pharmacy, Faculty of Natural Science, University of the Western Cape, Cape Town, South Africa. Email: okonjichristabel@gmail.com.

## Declaration of competing interest

None.

## Abbreviations

CFR: Case Fatality rate
COVID-19: Coronavirus 2019
DRC: Democratic Republic of the Congo Democratic Republic of the Congo
MPXV: Monkeypox virus
MPX: Monkeypox
WHO: World Health Organization
